# Acceptance of disability and its predictors among stroke patients in Taiwan

**DOI:** 10.1186/1471-2377-13-175

**Published:** 2013-11-14

**Authors:** Shan-Yun Chiu, Hanoch Livneh, Long-Lung Tsao, Tzung-Yi Tsai

**Affiliations:** 1 Department of Nursing, Dalin Tzuchi Hospital, The Buddhist Tzuchi Medical Foundation, No. 2, Minsheng Rd., Dalin Township, Chiayi 62247, Taiwan; 2 Rehabilitation Counseling Program, Portland State University, PO Box 751, Portland, OR 97207-0751, USA; 3 Department of Neurology, Dalin Tzuchi Hospital, The Buddhist Tzuchi Medical Foundation, No. 2, Minsheng Rd., Dalin Township, Chiayi 62247, Taiwan; 4 Department of Medical Research, Dalin Tzuchi Hospital, The Buddhist Tzuchi Medical Foundation, No. 2, Minsheng Rd., Dalin Township, Chiayi 62247, Taiwan; 5 Department of Nursing, Tzu Chi College of Technology, No. 880, Chien-Kuo Rd. Sec. 2, Hualien 97005, Taiwan; 6 Department of Environmental and Occupational Health, College of Medicine, National Cheng Kung University, No. 138, Sheng-Li Rd., Tainan 70428, Taiwan

**Keywords:** Acceptance of disability, Stroke, Taiwan

## Abstract

**Background:**

Modern medicine has increased the survival rate for stroke patients; however, the patient’s psychosocial adaptation after stroke onset may be related to the clinical outcomes. This study aimed to investigate patients’ acceptance of disability (AOD) and its predictors in stroke patients.

**Methods:**

This cross-sectional study used a purposive sampling method to recruit 175 stroke patients from a hospital in southern Taiwan. A structured questionnaire gathered data on respondent demographics and disease characteristics, and included the Chinese version of the AOD Scale-Revised. Factors associated with AOD were examined by a multiple linear regression analysis.

**Results:**

The mean AOD score was 71.72, which indicated a lower level of disease acceptance (range, 32-128). Our findings showed that patients who reported no religious beliefs, shorter disease duration, recurrent stroke episodes, and poorer physical functioning also reported lower levels of disability acceptance. These factors accounted for 38.2% of the variance in AOD among participants.

**Conclusions:**

The findings are beneficial to healthcare providers by identifying those stroke patients with predisposition of having lower disability acceptance, which could then facilitate the provision of appropriate rehabilitation interventions within six months after the diagnosis of stroke to support their adaptation process.

## Background

The World Health Organization (WHO) predicted that by 2020, stroke will be second only to ischemic heart disease as the leading cause of disability worldwide [1]. Given the complex symptoms and long duration of rehabilitation, the economic burden regarding stroke cannot be ignored. In the United States, more than 795,000 patients experience a new or recurrent stroke each year, accounting for direct and indirect healthcare costs totaling $21.8 billion and $65.5 billion, respectively [2]. Furthermore, the estimated medical expenses for one stroke patient per admission total approximately $20,000, which is three times the cost of care for a non-stroke patient [2].

Stroke does not only cause an enormous economic burden, but also triggers subsequent disability. For example, the risk of dementia for stroke patients is more than two-fold higher than for those without the disease [3]. A study by Koton et al. reported a 31.1% mortality rate for first-time stroke patients over a 3-year follow-up period [4]. The irreversible nature and unsatisfactory prognostic outcome associated with strokes often result in the development of psychiatric disorders, especially depression, in stroke patients. A previous study reported that the prevalence of depression after a stroke ranged from 13% to 72%, with a pooled estimate of 30% [5]. It should be noted that the diagnosis of depression, or of other mental disorders, in a stroke patient is associated with a 13% higher risk of mortality compared to a non-stroke patient, which emphasizes the urgent importance of mental health care for stroke patients [6].

An important intervention during rehabilitation focuses on helping patients with chronic disease accept their disabilities, learn how to cope and live with them, and adapt to the ensued physical and psychosocial sequelae [7]. Some researchers have suggested that one key factor that could account for such variation in psychosocial adjustment is acceptance of disability (AOD) [8,9]. In order to specifically measure the AOD, Linkowski developed a scale with 50 items that was based on the concepts concerning acceptance of loss proposed by Wright. According to Wright’s theory, AOD is associated with viewing one’s disability as a non-devaluating part of life through personal coping efforts and the realization that one can successfully overcome many of the imposed restrictions and limitations [10]. Recently, some studies involving patients with chronic diseases, such as those involving ostomy, spinal cord injury, insulin-dependent diabetes mellitus, and Ehlers-Danlos syndrome, have shown that higher levels of acceptance are associated with increased health-promoting behaviors, which could lower the risk of recurrence and medical complications [9,11,12]. Therefore, periodic screening of AOD for stroke patients should be integrated into routine care practice.

Within the Chinese culture, a stigma has been attached to the diagnosis of psychiatric disorders [13], so most of the literatures about stroke patients focused on survival rates [14], disease prevalence [15], or length of hospital stay [16]. Presently, no substantial discussions of AOD in stroke cases have been reported in the literature. Therefore, the aims of this study were to examine AOD and its related factors among patients with stroke in Taiwan, with the hope that these findings could be used as a reference for instituting appropriate psycho-physical interventions for Chinese stroke patients.

## Methods

### Study design and samples

This study applied a cross-sectional, correlational design with purposive sampling to recruit stroke patients from a hospital in Taiwan between June 2011 and July 2012. The inclusion criteria were as follows: (i) age ≥20 years old; (ii) absence of cognitive impairments and ability to express opinions in either Mandarin or Taiwanese; (iii) experienced a hemorrhagic or ischemic stroke >3 months before the study; and (iv) no family history of psychiatric disorders. Additionally, the sample size needed for this study was determined by the Cohen method [17], where α was set to 0.05, power to 0.8, and an effect size to 0.15, resulting in the need for a sample of at least 139 patients.

### Instruments

The Chinese Version of the Acceptance of Disability Scale-Revised (AODS-R) and an additional questionnaire that contained information on demographic and disease characteristics were used for data collection.

AODS-R was developed by Groomes & Linkowski in 2007 [10], and focused on the “enlargement of scope of values,” “subordination of physique,” “containment of disability effect,” and “transformation from comparative values to asset values”, all of which reflect Wright’s successful coping with disability paradigm. This revised scale consists of 32 items, which address individual attitude using a 4-point Likert scale ranging from 1 (strongly disagree) to 4 (strongly agree). Of the 32 items, 10 reflect positive values (#3, 6, 12, 16, 18, 21, 25, 28, 29, and 32), and the remaining 22 items reflect negative items. The full-scale range is from 32 to 128, with higher scores indicating a higher level of disease or disability acceptance. In terms of psychometric characteristics of this revised scale, a principal component analysis extracted four factors corresponding to the initial version. 42% of the total variance was explained, which indicates that the construct validity of the revised scale was acceptable. AODS-R scale has demonstrated good internal consistency among different groups of people with disabilities (Cronbach’s α between 0.91 and 0.93) [10,12].

The AODS-R has been translated into Chinese by Chiang et al. [8] in order to evaluate disease acceptance among Taiwanese chronic disease patients; these authors also examined the validity of the Chinese version by correlating it with the Taiwanese Depression Questionnaire (TDQ) [18] and reported a significant correlation coefficient of -0.45 (*P* < 0.01). As for internal consistency estimates of the Chinese version of the AODS-R, the findings of the corrected item-total score correlation were positive and statistically significant (*P* < 0.05), ranging from 0.28 to 0.55. Cronbach’s α derived from the present data yielded a coefficient of 0.94.

The second part of the questionnaire contained information on demographic and disease characteristics and was developed from previous literature and clinical experiences. The demographic data collected included gender, age, marital status, education, monthly income, living status, religion, and certain lifestyle factors, such as smoking, exercise, and sleep. Those who answered “currently” or “yes/past” to smoking were classified as smokers. Those who exercised ≥3 days per week were classified as regular exercisers. With respect to sleep, those patients who have awakened >2 times per week were classified as having a sleeping disturbance. The disease characteristics included the presence of chronic disease (ie, diabetes mellitus, hypertension, heart disease, or cancer), the number of strokes (first/recurrent), the type of stroke (hemorrhagic or ischemic), the duration of the stroke (in months), the body side of lesion, and activities of daily living (ADL); for the latter variable, it was determined using Barthel index. Scores on Barthel index ranged from 0 to 100 with higher scores indicating better functioning. All disease characteristics were obtained from the patients’ medical records.

### Data collection

This study was approved by the Institutional Review Board of Dalin Tzu Chi Hosiptal (B10001011). To ensure patients’ rights, the researchers explained the purpose of the study and procedures to the patients. Informed consent was obtained after the patient understood and agreed to participate in the study. During completion of the questionnaires, the researchers were available to answer any questions. For illiterate patients, the researchers read the questionnaires and recorded their answers. To ensure patients’ anonymity, the questionnaires were returned without any identifying information on them. Patients were also assured of complete confidentiality of all obtained data and were given the option to withdraw from the study at any time without any penalty.

### Data analysis

Data were analyzed using SPSS, version 17.0 for Windows (SPSS Inc., Chicago, IL, USA). Descriptive and inferential statistical analyses were conducted in accordance with the study aims and the nature of the variables. Descriptive analyses, including means and standard deviations (SD), were used to describe the distributions of the demographic data and disease characteristics. Intergroup difference of AOD level was tested using independent t-test for categorical variables and Pearson correlation for continuous variables, as appropriate. Variables that correlated significantly with the criterion measure (AOD level) were entered into multiple linear regression analysis to determine the significant predictors of disability acceptance.

To control for possible multicollinearity effects among AOD predictive variables, collinearity analyses, including tolerance value, variance inflation factor, and condition index, were performed before conducting the multivariate analysis. Also, the assumptions of normality, linearity, and homoscedasticity were tested. *p*-value was set at 0.05 for all statistical analyses.

## Results

### Demographic data and disease characteristics

A total of 175 stroke patients with a mean age of 66.23 years (SD = 11.24) were recruited during the period of data collection. The majority were males (71.4%), married (93.7%), had a low educational level (below 9^th^ grade) (79.4%), and cohabitating (87.4%). The majority of patients had a monthly income of New Taiwan Dollar (NTD) ≤ 30,000 (61.1%) and reported having religious beliefs (76.0%). Slightly less than a half reported sleep disturbances (45.1%), and approximately half were smokers (50.3%). Two-thirds of the patients engaged in regular exercise (66.3%). With regard to disease characteristics, the mean duration of stroke was 21.68 months (SD = 10.92), and the mean Barthel Index score was 75.09 (SD = 20.47). Most patients presented with an ischemic stroke (92.6%), a left side lesion (56.6%), a first stroke event (75.4%), and experienced other comorbidities (85.1%) (Table 1).

**Table 1:** Demographic and disease characteristics (N = 175)

**Variables**	**Mean ± SD**	***n *** **(%)**
Demographic data		
Age (yr)	66.23 ± 11.24	
Gender		
Male		125(71.4)
Female		50(28.6)
Marital status		
Single		11(6.3)
Married		164(93.7)
Educational level		
Low (<9th grade)		139(79.4)
High (>9th grade)		36(20.6)
Monthly income		
≦30000		107(61.1)
≧30001		68(38.9)
Living status		
Living alone		22(12.6)
Cohabitating		153(87.4)
Religious beliefs		
Yes		133(76.0)
No		42(24.0)
Sleep disturbance		
Yes		79(45.1)
No		96(54.9)
Cigarette smoking		
Yes		88(50.3)
No		87(49.7)
Regular exercise		
Yes		116(66.3)
No		59(33.7)
Disease characteristics		
Disease duration (mo)	21.68 ± 10.92	
Side of lesion		
Left		99(56.6)
Right		76(43.4)
Stroke event		
First		132(75.4)
Recurrent		43(24.6)
Comorbidity		
Yes		149(85.1)
No		26(14.9)
Stroke type		
Hemorrhagic		13(7.4)
Ischemic		162(92.6)
Barthel index		75.09 ± 20.47

### AOD scores

The mean score of AOD level was 71.72, with a SD of 11.27. Of the four AODS-R subscales, “enlargement of scope of values” had the highest standardized score (77.64), and “subordination of physique” had the lowest standardized score (43.95) (Table 2).

**Table 2:** Means and SD of the four AODS-R subscales (N = 175)

**Dimension**	**Mean**	**SD**	**Standardized score**^**(1)**^	**Rank**
Transformation from comparative values to asset values (9-36)	18.86	3.91	52.38	2
Enlargement of scope of values (9-36)	27.95	4.13	77.64	1
Containment of disability effect (9-36)	16.12	4.94	44.78	3
Subordination of physique (5-20)	8.79	1.96	43.95	4
Total scores (32-128)	71.72	11.27	56.03	

^**(1)**^Standardized scores = mean ÷ total score *100%.

### Correlations among demographic data, disease characteristics, and AOD scores

Table 3 shows the correlation between potential predictors and AODS-R. The results demonstrated that religious beliefs and sleep disturbance were related to the level of AOD (t = 2.55, *p* = 0.01; t = 2.89, *p* = 0.004, respectively). In terms of disease characteristics, all measured disease characteristics correlated with AOD score, with the exception of lesion side of body. For example, patients with first-ever stroke (t = 2.74; *p* = 0.007), no other chronic diseases (t = 2.87; *p* = 0.005), or higher daily activity levels (r = 0.49; *p* < 0.001) had higher AODS-R scores than those without these condition. On the other hand, disease duration correlated positively with AODS-R scores, indicating that higher AODS-R scores are related to longer disease duration (r = 0.2; *p* = 0.008).

**Table 3:** Relationships between demographic data, disease characteristics, and acceptance of disability (N = 175)

**Variables**	**AOD score**			
	**Mean**	**SD**	***t/r***	***p***
Demographic data				
Age (yr)			*r* = -0.03	0.73
Gender				
Male	71.73	11.59	*t* = -0.02	0.99
Female	71.70	12.14		
Marital status				
Single	71.70	11.78	*t* = 0.11	0.91
Married	72.09	11.34		
Educational level				
Low (below 9th grade)	71.53	11.99	*t* = -0.44	0.66
High (above 9th grade)	72.50	10.72		
Monthly income				
≦30000	70.88	12.12	*t* = -1.20	0.23
≧30001	73.06	11.01		
Living status				
Living alone	73.72	8.83	*t* = 1.08	0.29
Cohabitating	71.44	12.08		
Religious beliefs				
Yes	73.15	10.64	*t* = 2.55	0.01
No	67.21	13.83		
Sleep disturbance				
Yes	68.96	12.86	*t* = 2.89	0.004
No	74.00	10.21		
Cigarette smoking				
Yes	71.75	11.97	*t* = -0.03	0.98
No	71.70	11.53		
Regular exercise				
Yes	71.48	11.92	*t* = 0.38	0.70
No	72.20	11.39		
Disease characteristics				
Disease duration (mo)			*r* = 0.2	0.008
Side of lesion				
Left	71.42	12.22	*t* = 0.39	0.70
Right	72.19	11.11		
Stroke event				
First	73.02	11.05	*t* = 2.74	0.007
Recurrent	67.35	12.96		
Comorbidity				
Yes	70.68	11.32	*t* = 2.87	0.005
No	77.69	12.45		
Stroke type				
Hemorrhagic	65.84	12.78	*t* = 1.89	0.06
Ischemic	72.19	11.54		
Barthel index			*r* = 0.49	0.000

**Figure 1:**
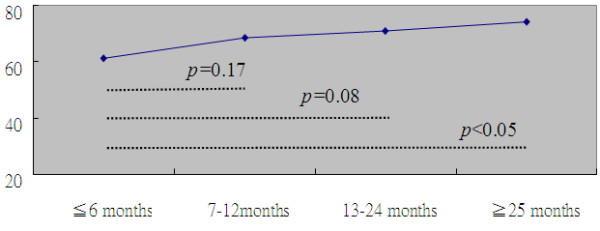
AODS-R scores among four groups within different disease duration.

Our study indicated a positive relationship between the duration of stroke and the AOD level, implying that those with longer time since stroke episode reported more positive disability acceptance. In the further analysis, we assessed the variation level of AOD within four different time periods (≤6 months, 7-12 months, 13-24 months, ≥25 months) and observed that the mean AODS-R score was the lowest in patients with disease duration ≤ 6 months. Mean AOD scores for the four time frames were 61.55 (SD = 9.17), 68.64 (SD = 15.70), 71.04 (SD = 12.79) and 74.19 (SD = 10.18), respectively (test for trend, *p* < 0.05) (Figure 1).

### Related factors affecting AOD

Multiple linear regression analysis was applied to examine which variables contributed to acceptance among stroke patients. Table 4 reveals that four predictor variables, including the presence of religious beliefs, disease duration, stroke event, and Barthel index scores, accounted for 38.2% of the variance in AODS-R scores. In other words, stroke patients with longer disease duration, religious beliefs, first-ever stroke and engaging in more daily activities reported higher levels of disability acceptance.

**Table 4:** Multivariate regression analysis predicting AODS-R scores among stroke patients (N = 175)

**Variables**	**β coef.**	***t***	***p***	**VIF**
Religious beliefs (Yes vs No)	4.61	2.66	<0.01	1.02
Disease duration	0.13	2.34	0.02	1.03
Stroke event (Recurrent vs First)	−4.05	−2.32	0.02	1.05
Barthel index	0.22	5.56	<0.01	1.21

【β coefficient with positive values indicate higher level of AOD; negative values reflect lower level of AOD】.

## Discussion

In this study of patients who experienced stroke, we found a raw mean AOD score of 71.72, and a standardized score of 56.03. Compared with AOD scores of patients with other chronic diseases assessed with the same tool (standardized score 62-87) [11,12,19,20], stroke patients had remarkably lower AOD scores. These lower scores likely reflect the overall disease burden, associated with chronic physical, cognitive and emotional impairments and the encountered economic and environmental barriers. For example, one study showed that the average hospital cost for a stroke patient is almost $20,000 dollars, which is nearly three times the cost for a non-stroke patient [21]; furthermore, disability-adjusted life-years (DALY) lost from strokes has become the fourth highest among all diseases worldwide [1]. Stroke unquestionably has an enormous impact on the patient, the family, and society at large, which may reduce AOD level to some extent.

Table 2 further demonstrated that lower AOD scores are primarily reflections of the “subordination of physique” and “containment of disability effects” domains, implying that the motor impairment may play a predominate role affecting AOD level among stroke patients. Recently, modified constraint-induced movement therapy (CIMT) was proven to be the most promising intervention to strengthen the mobility and fluency of movement in the affected limbs [22]. A randomized clinical trial conducted by Wolf and colleagues evaluated the effect of CIMT for 6 hours per day for two weeks on stroke patients, and reported that the CIMT participants had greater improvements than the those in the control group in the time of Wolf Motor Function Test (WMFT) and Motor Activity Log (MAL), by 34% and 43%, respectively [23]. Therefore, CIMT may be integrated into discharge planning in order to facilitate interventions, which will increase the self-care ability of stroke patients [24].

We found that patients who reported having religious beliefs had higher AOD scores than those who did not turn to religion. An earlier study had shown that religious beliefs was related to improved mental health [25]. People with religious beliefs may have more intangible psychological resources and support through spiritual guidance along with a reduction in psychological stress. This, in turn, can enhance the immune system and reduce symptoms associated with disease [26]. But our result is inconsistent with the findings of Chao et al*.*[19], who reported the null association between AOD level and religious beliefs among colorectal cancer patients. For most people, the diagnosis of cancer is psychologically associated with incurable illness or death, and often results in a significant psychological impact on people of Asian descent [27]. Therefore, the influence of psychological and spiritual support from religion may not be so apparent for cancer patients.

The duration of disease was positively correlated with AOD, which is consistent with a previous argument that AOD research is more meaningful when conducted in the context of patients with chronic diseases [9,19]. We speculate that stroke patients with a longer duration of disease have gradually learned to accept the consequences and future implications of their disability and thus have higher AOD scores. It is notable that patients whose duration of disease was ≤ 6 months had the lowest AOD scores. We recommend that healthcare providers should pay greater attention to newly-diagnosed stroke patients by establishing a set of rehabilitation care process for evaluating their signs of psycho-physical status to facilitate early referral for further therapeutic interventions [28].

The findings of this study also indicate that AOD scores of recurrent stroke patients were lower than those of first-time stroke patients. This finding is consistent with a previous argument that secondary stroke patients tend to become more distressed and therefore experience more negative emotions, such as anxiety, posttraumatic stress and depression [29,30]. Recurrent strokes often result in aggravated cerebral leukoaraiosis, and thus worsen limb dysfunction and intellectual impairment [31,32]. Furthermore, recurrent stroke patients were found to have higher monthly medical expenditures than those with newly-diagnosed stroke by 375 US dollars [33], which may result in lower levels of AOD because of heavier economic burdens and concerns about their future.

Findings from our study also suggest that there was a significant positive correlation between mobility and AOD scores in stroke patients, echoing previous research findings about physical mobility and emotional distress [11,34]. Patients with greater mobility are more likely to be successfully integrated into social support networks, maintain good interpersonal relationships, require less help from others in daily activities, and thus have higher AOD scores. This finding is inconsistent with findings of other studies [35,36], however, this may be partially due to differences in sample size and age distribution of participants. Previous studies all used the Barthel index to evaluate patient mobility and to analyze its relationship with depressed mood by surveying small numbers of patients (n ≤ 50) [35,36]. Such small sample sizes may provide insufficient statistical power in multivariate analysis and thus fail to clarify the true relationships among variables. Furthermore, our subjects (≈ 67 years on average) were older than previously studied cases (46.01 and 51.40, respectively). It can be argued that with different physical, and possibly cognitive, functioning, the impact of impaired mobility on AOD would be different.

Although the results of this study provide for several important clinical and research implications, a few limitations must be acknowledged. First, the study subjects were recruited from a single hospital, which may limit the generalizability of the findings. Future studies should use larger samples, including random or stratified sampling methods of data collection, to improve the sample representation. Nonetheless, we calculated a required sample size analysis to ensure statistical power before embarking on the study, and thus, the sample size used in this study may be considered satisfactory for exploring the predictors associated with AOD level in stroke patients. Second, since this study used a cross-sectional design, we cannot infer causality from our findings. A longitudinal research design is needed to examine any causal relationships among the factors assessed in this study. Furthermore, there is a need to investigate the relationship between AOD level and subsequent clinical manifestations. Third, sleep disturbance and religious beliefs were only assessed with a single-item question, and therefore, their psychometric characteristic may be suspect and, accordingly, must be interpreted cautiously. Despite these methodological concerns, to our knowledge, this was the first study to assess the level of AOD among Taiwanese stroke patients, a fact which can be used as a future reference in developing the timely therapeutic regimen.

## Conclusion

In conclusion, advances in medical techniques have extended survival of chronically-ill patients; however, cognitive, emotional and physical distress, triggered by both condition’s sequelae and its associated treatment modalities, may easily disrupt patients’ lives and induce further psychiatric symptoms. This study found that the mean AOD score among Taiwanese stroke patients was 71.72, suggesting a lower level of disability acceptance than that reported in earlier studies. Among stroke patients, those with no religious beliefs, with shorter duration of disease, with recurrent stroke, or with poor ability to perform daily activities, reported lower AODS-R scores. Healthcare providers should consider instituting an appropriate rehabilitation care procedure for stroke patients, supplemented by a clinically-validated and psychometrically sound assessment tool. Actually, it is imperative to help patients better psychologically adapt to their disease and possibly as important as improving the survival rate of patients with the chronic and life threatening disease.

## Abbreviations

AOD: Acceptance of disability; AODS-R: Acceptance of disability scale-revised; TDQ: Taiwanese depression questionnaire; SD: Standard deviations; NTD: New Taiwan dollar; DALY: Disability-adjusted life-years.

## Competing interests

No conflict of interest has been declared by the authors.

## Authors’ contributions

SYC was responsible for data collection and participated in providing comments on the manuscript drafts. HL contributed to the interpretation of data and providing comments on the final draft of the manuscript. LLT provided administrative support and comments on the manuscript drafts. TYT was responsible for the study conception, design, data analysis, and drafting of the work. All authors read and approved the final manuscript.

## Pre-publication history

The pre-publication history for this paper can be accessed here:

http://www.biomedcentral.com/1471-2377/13/175/prepub
